# Supplementary material for: Chlorhexidine Mucoadhesive Buccal Tablets: The Impact of Formulation Design on Drug Delivery and Release Kinetics Using Conventional and Novel Dissolution Methods
Source: Pharmaceuticals (Basel). 2021 May 23;14(6):493. doi: 10.3390/ph14060493 (PMC8224615; doi:10.3390/ph14060493)
Supplement: Supplementary file 1 [file pharmaceuticals-14-00493-s001.zip › pharmaceuticals-1212419-supplementary.pdf]

Supplementary Materials

Figure S1 shows the morphology of granules after mixing in the water bath for 1-3 minutes at 57°C.

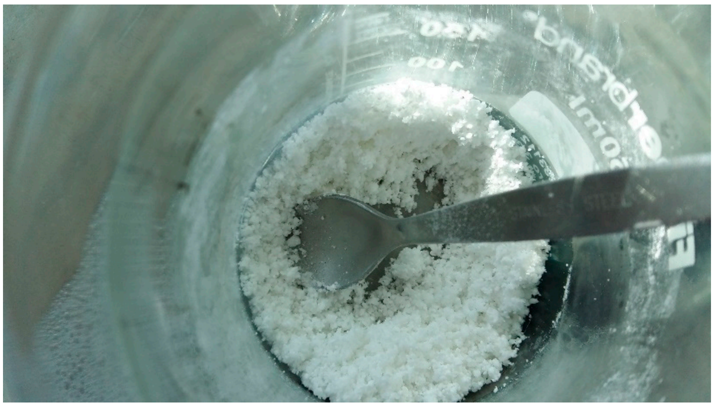

Figure S1 Melt Granules-S2 formulation

Swelling Index

The swelling index of CHD tablets using different polyols is shown in Figure S2. The swelling profiles show a rapid initial swelling during the first 30 minutes followed by a slower rate of swelling for the following 90 minutes. S4, M4 and X4 have a SI of 3 after 2 hours. However, S1, M1 and X1 no difference has been reported ($p>0.5$) in swelling index, with values of 3.8, 4.2 and 4.4, respectively. These differences are not statistically significant based on two-way ANOVA analysis.

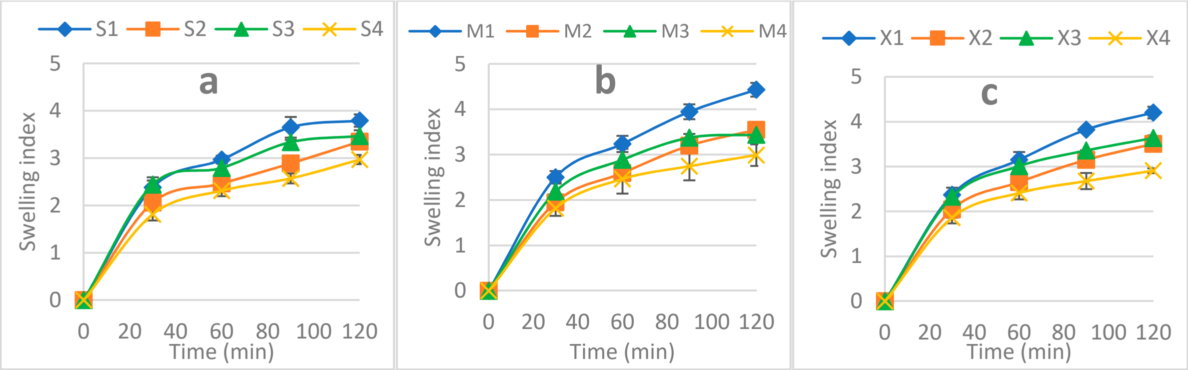

Figure S2 Swelling index of CHD tablets a-sorbitol, b-mannitol and c-xylitol formulations, at 37 °C and for two hours. Data are expressed as mean ± SD, n = 3.

Scanning Electron Microscopy (SEM)

Aqueous media swollen tablets were prepared by placing each tablet into a 5 ml test tube containing 0.5 ml of water. The tablets were freeze-dried using a Beta 1-8 LSC Freeze Dryer (Christ, UK) after being hydrated. Prior to SEM analysis, the tablets were cut in half. Images were acquired using SEM (Zeiss Evo® 50EP SEM, Germany).

All formulations successfully formed a porous structure (Figure S3), although formulations with a higher P407 ratio were very fragile after freeze-drying, and the pores were damaged when the tablets were cut.

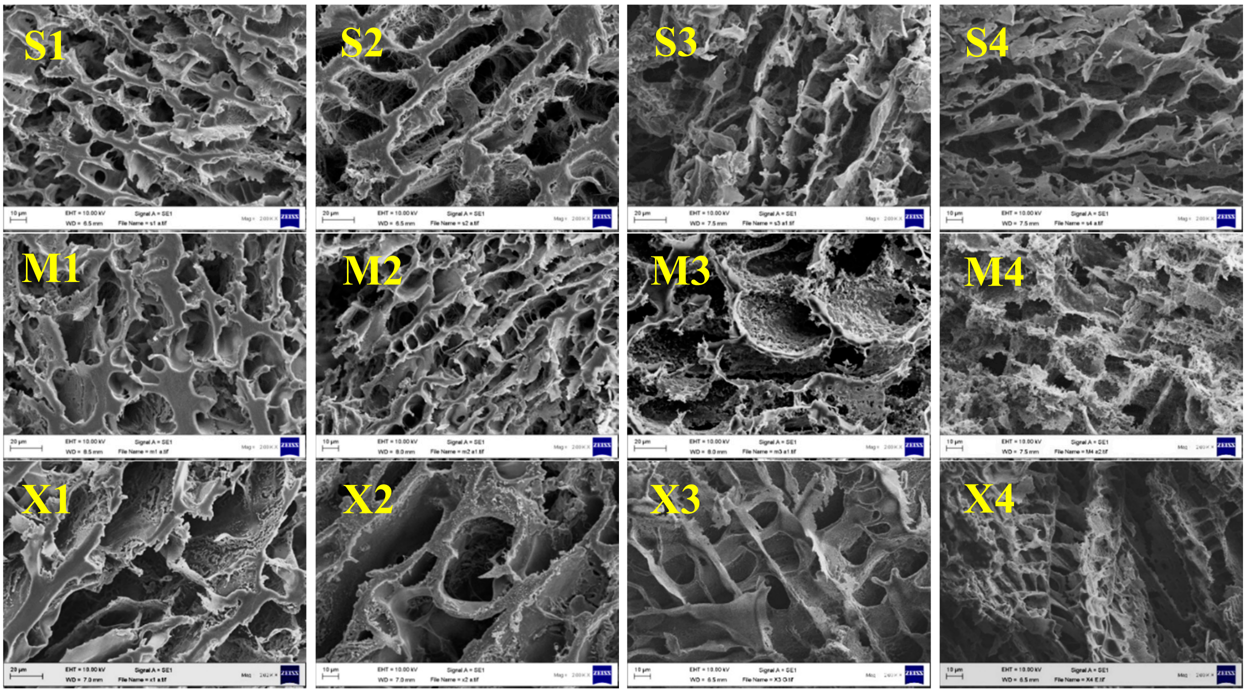

Figure S3 SEM images showing the porous structure of the freeze-dried swollen tablets containing sorbitol, mannitol and xylitol, 2000x.

Drug polymer interaction:

Fourier Transform Infrared Spectroscopy (FTIR)

FTIR was used to investigate the possibility of interaction between the polymers, polyols and CHD. Spectra were obtained using a Bruker Alpha spectrometer (Germany) and ranged from 4000 to 400 cm^{-1}.

The FTIR spectra for all tablet contents and physical mix (PM), granules (Gran) and for S4, M4 and X4 are shown in Figure S4.

By comparing the spectra of the physical mixes and the granules of each formulation, no difference was observed between each spectrum. Consequently, it is concluded that there is no interaction caused by the granulation process. Moreover, the spectra of all formulations showed dominant P407 peaks, characterised principally by peaks at 2875 cm^{-1} (CH stretching) and 1094 cm^{-1} (CO stretching). This is attributed to the steric hindrance or stabiliser effect of P407 which constitute approximately 50% of the tablet weight [1].

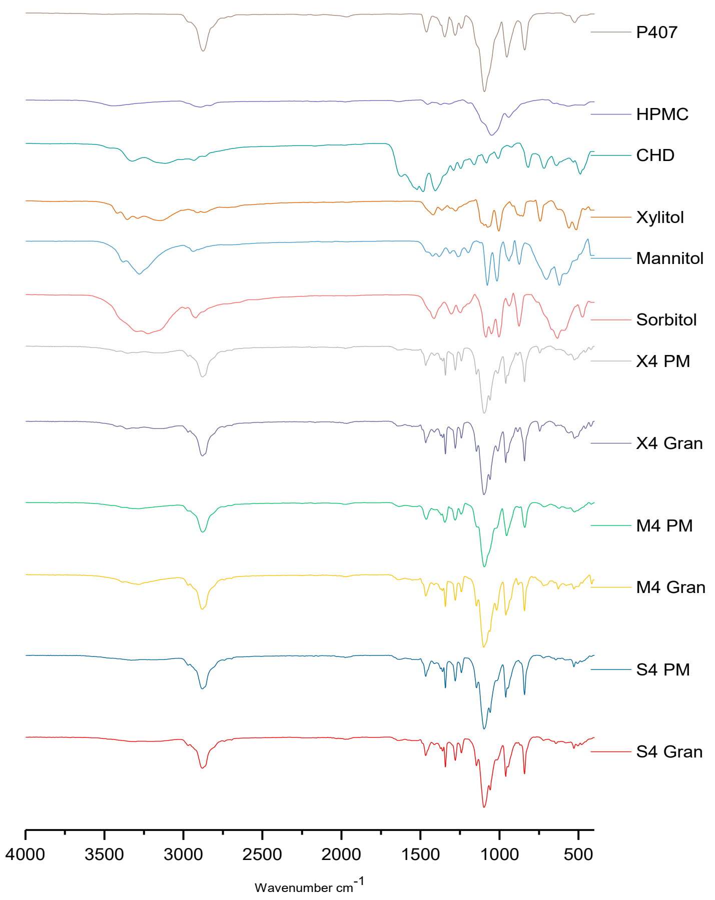

Figure S4 FTIR spectra of tablet excipients, CHD, S4, M4 and X4 physical mix (PM) and granules (Gran).

Differential Scanning Calorimetry (DSC)

DSC analysis was applied to the raw materials, CHD, HPMC, P407, Sorbitol, Mannitol and Xylitol, S4, M4 and X4 formulations, physical mix, granules and tablets to investigate any possible interactions between CHD and the excipients during the preparation of the tablets. Samples of 5.0 ± 0.2 mg each were heated in an aluminium pan under a nitrogen flow rate of 40 ml/min from 25°C to 300°C at a scan rate of 10°C/min. The analysis was performed using a Mettler Toledo DSC823e (Switzerland). As shown in Figure S5, HPMC is amorphous, whereas the rest of the ingredients are crystalline with distinctive melting peaks. The thermograms indicate no interaction during granulation and tablet pressing. Moreover, the disappearance of the melting peak of CHD in the physical mixes, granules and tablets suggests the dissolution of CHD in P407, the latter having a low melting point of ~70°C. A summary of the melting peaks is presented in Table S1

Table S1 Melting peaks obtained from the DSC thermograms of CHD, the excipients, S4, M4 and X4 PM, Granules and Tablets.

Material	Melting Peak (°C)	S1 PM	S1 G	S1 T	M1 PM	M1 G	M1 T	X1 PM	X1 G	X1 T
CHD	156.79	-	-	-	-	-	-	-	-	-
P407	56.79	56.97	57.23	57.38	56.83	57.24	57.43	56.65	57.08	57.07
Sorbitol	99.80	99.54	99.62	99.19	-	-	-	-	-	-
Mannitol	166.31	-	-	-	165.62	165.24	165.10	-	-	-
Xylitol	94.06	-	-	-	-	-	-	93.95	93.32	93.54

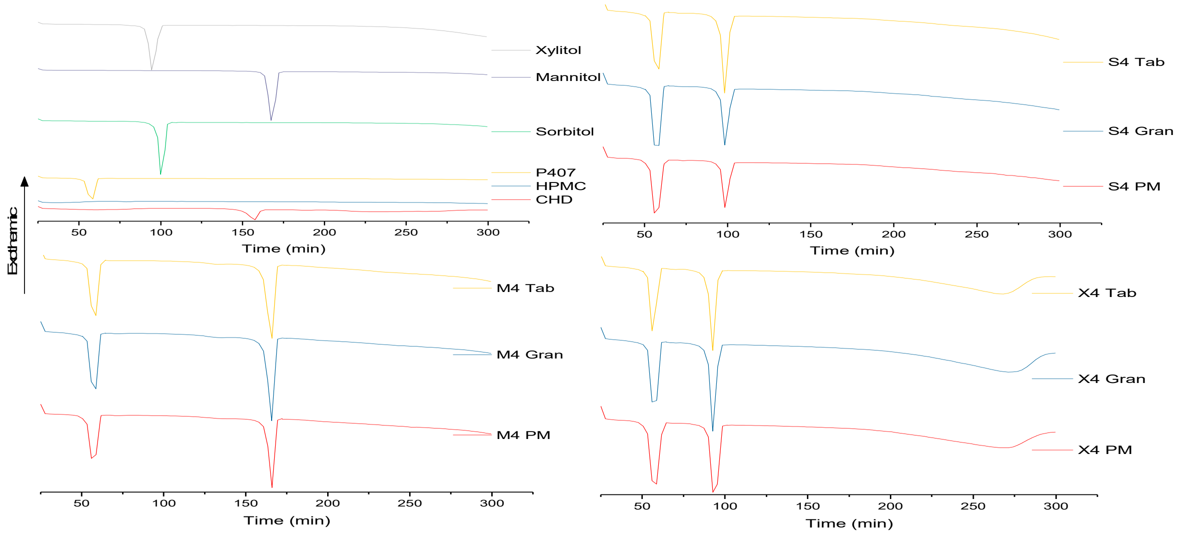

Figure S5 DSC thermograms of tablet excipients, CHD, S4, M4 and X4 physical mix (PM) and granules (Gran) and tablets (Tab).

Non-Cumulative Drug Release

Figure S6 shows drug release using the CFR method at 1ml/min for two hours. The release is expressed in µg/ml. The polyols had no impact at low P407 ratios. However, they had an effect on drug release from S4, M4 and X4 when compared with S3, M3 and X3. This can be explained by the solubility of the polyols.

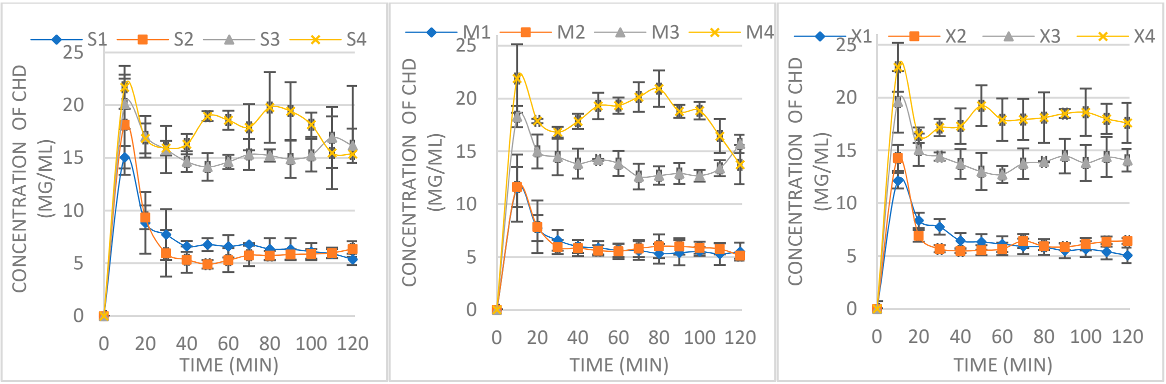

Figure S6 Non-cumulative CHD release using CFR at 1ml/min. Data are expressed as mean concentration ± SD, n = 3.

Kinetics of drug release

Hopfenberg model

$$F = 100 \times [1 - (1 - K_{Bt} \times t)^n] \qquad 1$$

Where F is the fraction percent of drug released at time t.

K_{Bt} is the combined constant, and it can be obtained from Equation 2

$$K_{Bt} = \frac{K_0}{(C_0 \times a_0)} \qquad 2$$

C_0 is the initial concentration of drug in the matrix, and a_0 is the initial radius for a sphere or a cylinder or the half thickness for a slab; *n* is 1, 2, and 3 for a slab, cylinder, and sphere, respectively.

The coefficients determination (R^2) for first order, Higuchi and Hixon Crowell models are presented in Table S2. They are lower than for zero order, Korsmeyer Peppas and Hopfenberg models.

Table S2 Drug release kinetics using first order, Higuchi and Hixon Crowell models for CHD formulations.

	Apparatus 1			CFR		
	First order (R^2)	Higuchi (R^2)	Hixon Crowell (R^2)	First order (R^2)	Higuchi (R^2)	Hixon crowell (R^2)
**S1**	0.991 ± 0.009	0.965 ± 0.003	0.988 ± 0.014	0.980 ± 0.024	0.966 ± 0.020	0.974 ± 0.031
**S2**	0.984 ± 0.003	0.943 ± 0.008	0.998 ± 0.006	0.952 ± 0.026	0.973 ± 0.018	0.942 ± 0.031
**S3**	0.991 ± 0.003	0.982 ± 0.009	0.986 ± 0.006	0.970 ± 0.013	0.931 ± 0.017	0.985 ± 0.009
**S4**	0.980 ± 0.009	0.953 ± 0.015	0.992 ± 0.005	0.944 ± 0.005	0.921 ± 0.005	0.970 ± 0.003
**M1**	0.988 ± 0.013	0.950 ± 0.025	0.998 ± 0.006	0.990 ± 0.006	0.963 ± 0.008	0.985 ± 0.007
**M2**	0.991 ± 0.001	0.956 ± 0.008	0.989 ± 0.004	0.989 ± 0.013	0.955 ± 0.018	0.985 ± 0.016
**M3**	0.960 ± 0.009	0.923 ± 0.005	0.980 ± 0.007	0.984 ± 0.002	0.939 ± 0.007	0.994 ± 0.002
**M4**	0.965 ± 0.009	0.942 ± 0.011	0.982 ± 0.007	0.943 ± 0.005	0.925 ± 0.004	0.970 ± 0.004
**X1**	0.983 ± 0.020	0.979 ± 0.011	0.971 ± 0.039	0.988 ± 0.002	0.967 ± 0.004	0.983 ± 0.003
**X2**	0.994 ± 0.001	0.957 ± 0.008	0.993 ± 0.004	0.986 ± 0.005	0.955 ± 0.008	0.982 ± 0.007
**X3**	0.980 ± 0.013	0.939 ± 0.014	0.991 ± 0.008	0.979 ± 0.008	0.935 ± 0.011	0.990 ± 0.005
**X4**	0.972 ± 0.007	0.953 ± 0.008	0.989 ± 0.004	0.947 ± 0.002	0.924 ± 0.006	0.972 ± 0.001

Reference:

1. Beck-Broichsitter, M.; Bohr, A.; Ruge, C.A. Poloxamer-Decorated Polymer Nanoparticles for Lung Surfactant Compatibility. *Mol. Pharm.* **2017**, *14*, 3464–3472, doi:10.1021/acs.molpharmaceut.7b00477.
